# High Discrepancy in Abdominal Obesity Prevalence According to Different Waist Circumference Cut-Offs and Measurement Methods in Children: Need for Age-Risk-Weighted Standardized Cut-Offs?

**DOI:** 10.1371/journal.pone.0146579

**Published:** 2016-01-08

**Authors:** Alice Monzani, Anna Rapa, Flavia Prodam, Nicola Fuiano, Giuliana Diddi, Antonella Petri, Simonetta Bellone, Gianni Bona

**Affiliations:** 1 Division of Pediatrics, Department of Health Sciences, Università del Piemonte Orientale, Novara, Italy; 2 Pediatric Unit, Prevention and Public Health, ASL FG, Foggia, Italy; University, ITALY

## Abstract

**Background:**

Waist circumference (WC) is a good proxy measure of central adiposity. Due to the multiplicity of existing WC cut-offs and different measurement methods, the decision to use one rather than another WC chart may lead to different prevalence estimates of abdominal obesity in the same population. Aim of our study was to assess how much the prevalence of abdominal obesity varies in Italian schoolchildren using the different available WC cut-offs.

**Methods:**

We measured WC at just above the uppermost lateral border of the right ilium in 1062 Italian schoolchildren aged 7–14 years, 499 living in Northern Italy and 563 in Southern Italy. Abdominal obesity was defined as WC ≥90^th^ percentile for gender and age according to nine WC charts.

**Results:**

We found an extremely high variability in the prevalence of abdominal obesity detected in our study-populations according to the different WC charts, ranging in the overall group from 9.1% to 61.4%. In Northern Italy children it varied from 2.4% to 35.7%, and in Southern ones from 15.1% to 84.2%.

**Conclusions:**

On the basis of the chosen WC cut-offs the prevalence of abdominal obesity varies widely, because percentile-charts are strongly influenced by the population status in a particular moment. A further rate of variability may lay on the site of WC measurement and on the statistical method used to calculate WC cut-offs. Risk-weighted WC cut-offs measured in a standardized anatomic site and calculated by the appropriate method are needed to simply identify by WC measurement those children at high risk of cardio-metabolic complications to whom specific and prompt health interventions should be addressed.

## Introduction

The prevalence of paediatric overweight and obesity is increasing worldwide and this epidemic is cause of concern due to the association of obesity with morbidity even in childhood and to the high persistency of obesity in adulthood [1,2]. A prompt detection of fatness excess by general paediatricians may be the key to prevent serious metabolic and cardiovascular consequences.

Body mass index (BMI) has commonly been used to identify overweight and obesity in children and adolescents. However, it is an indicator of general adiposity and gives no indication about fat distribution [3]. During childhood and adolescence, it has been reported that abdominal obesity (AO) is an important predictor for several cardiovascular diseases [4,5]. Furthermore, AO is a critical component of the paediatric definitions of metabolic syndrome [6,7]. Waist circumference (WC) has been reported to be a good proxy measure of both visceral and subcutaneous adiposity, easy to be performed in clinical practice and associated with clustered cardiovascular risks in childhood [5,8]. Reference percentiles based on nationally representative samples do not already exist for all nations. Moreover, the available cut-offs arise from WC values measured in different body sites, e.g. at just above the uppermost lateral border of the right ilium [9–12] or at the midpoint between the lowest rib and the iliac crest [13–17]. So far, the decision to use one rather than another WC chart may lead to different prevalence estimates of AO in the same population. The comparison among rates of prevalence obtained by different WC charts may be questionable. Hence, the aim of our study was to assess how much AO prevalence varies in Italian schoolchildren using the available WC cut-offs.

## Materials and Methods

The study was carried out on a total of 1062 schoolchildren aged between 7 and 14 years, 499 children (M/F = 243/256) living in a centre of Northern Italy (Piemonte Region, Novara, 105.000 inhabitants) and 563 children (M/F = 306/257) living in a centre of Southern Italy (Puglia Region, San Marco in Lamis, 15.000 inhabitants). In Novara, children came from 4 out of 7 primary and secondary school of the city, whereas in San Marco in Lamis they represented the whole primary and secondary school population (3 schools). We obtained written informed consent from the parents, caretakers, or guardians on behalf of all the children enrolled in our study. Also the children provided their verbal assent, which was not recorded for the refusal of parents or caretakers. Signed informed consent forms was kept in a locked file cabinet, unavailable to anyone except those individuals outlined in the approved study. The study was approved by the Local Ethical Committees of Novara and Foggia, which both approved the consent procedure.

Anthropometric measurements were all collected by the same well trained operator (N.F.) and performed in duplicate for each child, wearing light indoor clothing and without shoes: weight (measured to the nearest 100 g with a spring scale tested daily for accuracy and calibrated against a set of standard weights—Salus, Inc., Italy), height (measured with a standard laboratory stadiometer—Holtain, Wales, UK- to the nearest 0.5 cm during maximal expiration) and WC (using a flexible inextensible tape—Seca, Milano, Italy—at just above the uppermost lateral border of the right ilium, at the end of a normal expiration). BMI was calculated as the ratio between weight (kg) and squared height (m^2^). Weight-SDS, height-SDS and BMI-SDS were calculated according to Cacciari et al. [18]. BMI categories (underweight, normal weight, overweight, obesity and morbid obesity) were defined according to BMI cut-offs of the International Obesity Task Force [19]. AO was defined as WC ≥ 90^th^ percentile for gender and age according to nine available charts [9–17]. We selected only the charts including the 90^th^ percentile cut-off values for children in our age range (7–14 years). All charts reported tables with the 90^th^ percentile value for males and females by one-year interval of age and WC of each child was checked on them. Waist-to-height ratio (WHtR) was calculated as the ratio of WC (cm) and height (cm), and a cut-off of 0.5 was used to define AO [20].

Pubertal evaluation was not performed for the refusal of the ethical committees due to the school setting of the study.

Descriptive statistics were expressed as median and range values. Prevalence of BMI categories and AO was reported as percentage and 95% confidence interval. Two-tailed chi-square or Fisher exact test were used to evaluate differences of prevalence, as appropriate. The statistical analysis was performed using STATISTICA version 6.1 (Stat Soft, Inc., Tulsa, OK).

## Results

Clinical characteristics of all schoolchildren according to gender and living area are reported in Table 1.

**Table 1:** Clinical characteristics of all schoolchildren according to gender and living area.

	Overall			Northern Italy (Piemonte)			Southern Italy (Puglia)		
	M	F	Total	M	F	Total	M	F	Total
	(n = 549)	(n = 513)	(n = 1062)	(n = 243)	(n = 256)	(n = 499)	(n = 306)	(n = 257)	(n = 563)
Age (years)	10.5	10.5	10.5	11.4	11.1	11.2	9.9	9.8	9.8
	(6.8–14)	(6.7–13.4)	(6.7–14)	(8.2–14)	(8.1–13.4)	(8.1–14)	(6.8–13)	(6.7–12.8)	(6.7–13)
Weight-SDS	0.17	0.03	0.10	-0.02	-0.04	-0.03	0.32	0.12	0.19
	(-3.24–3.32)	(-4.16–3.73)	(-4.16–3.73)	(-3.13–3.32)	(-2.08–3.73)	(-3.13–3.73)	(-3.24–3.16)	(-4.16–2.77)	(-4.16–3.16)
Height-SDS	0.19	0.30	0.23	0.22	0.41	0.31	0.11	0.12	0.12
	(-2.75–3.10)	(-2.81–3.30)	(-2.81–3.30)	(-2.75–3.10)	(-2.13–3.30)	(-2.75–3.30)	(-2.43–3.02)	(-2.81–2.76)	(-2.81–3.02)
BMI-SDS	0.19	-0.04	0.06	-0.11	-0.27	-0.21	0.34	0.13	0.27
	(-4.63–2.88)	(-4.49–3.17)	(-4.63–3.17)	(-3.37–2.88)	(-3.05–3.17)	(-3.37–3.17)	(-4.63–2.74)	(-4.49–2.54)	(-4.63–2.74)
WC (cm)	68	67	67.5	64.5	62.5	63.0	71	72	71
	(44–106)	(43.5–103.5)	(44–106)	(44–101)	(46.5–102)	(44–102)	52.5–106)	(51–103.5)	(51–106)
Underweight	5.8%	6.6%	6.2%	8.2%	8.2%	8.2%	3.9%	5.1%	4.4%
	(4.2–8.1)	(4.8–9.1)	(4.9–7.8)	(5.4–12.4)	(5.4–12.2)	(6.1–11)	(2.3–6.7)	(3–8.5)	(3–6.5)
Normal weight	56.5%	63.4%	59.8%	58.8%	69.5%	64.3%	54.6%	57.2%	55.8%
	(52.3–60.6)	(59.1–67.4)	(56.8–62.7)	(52.6–64.9)	(63.6–74.8)	(60–68.4)	(49–60.1)	(51.1–63.1)	(51.6–59.8)
Overweight	24.6%	22.2%	23.4%	23%	16.4%	19.6%	25.8%	28%	26.8%
	(21.2–28.4)	(18.8–26)	(21–26.1)	(18.2–28.7)	(12.4–21.4)	(16.4–23.4)	(21.2–31)	(22.9–33.8)	(23.3–30.6)
Obesity	9.8%	5.3%	7.6%	7%	4.3%	5.6%	12.1%	6.2%	9.4%
	(7.6–12.6)	(3.6–7.5)	(6.2–9.4)	(4.4–10.9)	(2.4–7.5)	(3.9–8)	(8.9–16.2)	(3.9–9.9)	(7.3–12.1)
Morbid Obesity	3.3%	2.5%	2.9%	2.9%	1.6%	2.2%	3.6%	3.5%	3.6%
	(2.1–5.1)	(1.5–4.3)	(2.1–4.1)	(1.4–5.8)	(0.6–3.9)	(1.2–3.9)	(2–6.3)	(1.9–6.5)	(2.3–5.4)
Abdominal Obesity by WHtR ≥ 0.5	42.4%	38.6%	40.6%	22.2%	10.9%	16.4%	58.5%	66.2%	62%
	(38.3–46.7)	(34.4–43)	(37.6–43.6)	(17.3–28.1)	(7.5–15.6)	(13.4–20)	(52.7–64)	(60–71.8)	(57.8–66)

Data are expressed as median (range) for age, weight-SDS, height-SDS, BMI-SDS and WC and as percentage (95% confidence interval) for BMI categories according to BMI cut-offs of the International Obesity Task Force (ref.[18]).

There were no significant differences between males and females in overall, Northern and Southern Italian schoolchildren for age, weight-SDS, height-SDS and BMI-SDS. Median WC was significantly higher in males than in females both in overall (68 vs 67 cm, p = 0.03) and in Northern Italy children (64.5 vs 62.5 cm, p = 0.0006). The prevalence of normalweight status was significantly higher in females than in males both in Northern Italy (69.5% vs. 58.8%, p = 0.02) and in overall children (63.4% vs. 56.5%, p = 0.03). The prevalence of obesity was significantly higher in males than in females both in Southern Italy (12.1% vs. 6.2%, p = 0.03) and in overall children (9.8% vs. 5.3%, p = 0.007).

The prevalence of AO according to different WC cut-offs is reported in Table 2.

**Table 2:** Prevalence of abdominal obesity according to different WC cut-offs and measurement methods.

	Overall			Northern Italy (Piemonte)			Southern Italy (Puglia)		
WC ≥90^th^ percentile according to:	M	F	Total	M	F	Total	M	F	Total
	(n = 549)	(n = 513)	(n = 1062)	(n = 243)	(n = 256)	(n = 499)	(n = 306)	(n = 257)	(n = 563)
***WC measurement at just above the uppermost lateral border of the right ilium***									
NHANES III cut-offs [9]	25.1%	26.5%	25.8%	10.7%	8.6%	9.6%	36.6%	44.4%	40.1%
	(21.7–28.9)	(22.9–30.5)	(23.3–28.5)	(7.4–15.2)	(5.7–12.7)	(7.3–12.5)	(31.4–42.1)	(38.4–50.5)	(36.2–44.2)
Italian cut-offs [10]^a^	11.8%	6.2%	9.1%	4.1%	0.8%	2.4%	18%	11.7%	15.1%
	(9.4–14.8)	(4.5–8.7)	(7.5–11)	(2.3–7.4)	(0.2–2.8)	(1.4–4.2)	(14.1–22.7)	(8.3–16.2)	(12.4–18.3)
Cypriot cut-offs [11]	23.7%	26.3%	25%	12.3%	8.2%	10.2%	32.7%	44.4%	38%
	(20.3–27.4)	(22.7–30.3)	(22.4–27.6)	(8.8–17.1)	(5.4–12.2)	(7.9–13.2)	(27.7–38.1)	(38.4–50.5)	(34.1–42.1)
Australian cut-offs [12]	49.4%	46%	47.7%	30%	18.8%	24.2%	64.7%	73.2%	68.6%
	(45.2–53.5)	(41.7–50.3)	(44.7–50.7)	(24.6–36.1)	(14.4–24)	(20.7–28.2)	(59.2–69.8)	(67.4–78.2)	(64.6–72.3)
***WC measurement at the midpoint between the lowest rib and the iliac crest***									
British cut-offs [13]	61.4%	61.4%	61.4%	37.4%	34%	35.7%	80.4%	88.7%	84.2%
	(57.2–65.4)	(57.1–65.5)	(58.4–64.3)	(31.6–43.7)	(28.5–40)	(31.6–40)	(75.6–84.5)	(84.3–92)	(80.9–87)
Dutch cut-offs [14]	51.5%	48.5%	50.1%	30.5%	19.5%	24.8%	68.3%	77.4%	72.5%
	(47.4–55.7)	(44.2–52.9)	(47.1–53.1)	(25–36.5)	(15.1–24.8)	(21.3–28.8)	(62.9–73.3)	(71.9–82.1)	(68.6–76)
Polish cut-offs [15]	39.3%	43.1%	41.1%	24.3%	16.8%	20.4%	51.3%	69.3%	59.5%
	(35.3–43.5)	(38.9–47.4)	(38.2–44.1)	(19.3–30)	(12.7–21.9)	(17.1–24.2)	(45.7–56.9)	(63.4–74.6)	(55.4–63.5)
Turkish cut-offs [16]^b^	48.1%	53.4%	50.7%	28.8%	25%	26.9%	63.4%	81.7%	71.8%
	(43.9–52.3)	(49.1–57.7)	(47.7–53.7)	(23.5–34.8)	(20.1–30.6)	(23.2–30.9)	(57.9–68.6)	(76.5–86)	(67.9–75.3)
Hong Kong Chinese cut-offs [17]	44.4%	57.7%	50.8%	28%	30.5%	29.3%	57.5%	84.8%	70%
	(40.3–48.6)	(53.4–61.9)	(47.8–53.8)	(22.7–33.9)	(25.2–36.4)	(25.4–33.4)	(51.9–62.9)	(79.9–88.7)	(66.1–73.6)

Prevalence is expressed as percentage and 95% confidence interval.

^a^ Cut-offs from a survey limited to Pescara, a town in a region of Central Italy

^b^ Cut-offs from a survey limited to Kayseri Province in Central Turkey.

In the overall group the prevalence of AO ranged from 9.1% to 61.4% according to the used WC cut-offs. In particular, it ranged from 9.1% to 47.7% when we used charts measuring WC at just above the uppermost lateral border of the right ilium, as we did. The prevalence ranged from 41.1% to 61.4% when we used charts measuring WC at the midpoint between the lowest rib and the iliac crest.

In Northern Italy children AO prevalence varied from 2.4% to 24.2% and in Southern ones from 15.1% to 68.6% using charts measuring WC at just above the uppermost lateral border of the right ilium, as we did.

The prevalence of overweight + obesity + morbid obesity according to BMI was 27.5% (137/499) in Northern Italy schoolchildren and 39.8% (224/563) in Southern ones (Table 1).

The prevalence of AO according to WHtR ratio was 16.4% (82/499) in Northern Italy schoolchildren and 62.0% (349/563) in Southern ones (Table 1).

Analysing differences in WC values between Northern and Southern Italy schoolchildren depending on BMI categories, we found that median (range) WC values were significantly higher (p<0.0001) in Southern schoolchildren than in Northern ones in underweight [58.2 (51–70.6) vs. 54 (44–62) cm], normalweight [67.25 (52.4–84.2) vs. 62 (46.5–76.5) cm] and overweight categories [77.7 (57.3–96.2) vs. 73 (61–91) cm].

## Discussion

The main result of our study was the wide variability in the prevalence of AO detected in our study-populations according to nine different WC charts, ranging from 9.1% to 61.4%. Similar findings were drawn by a recent work assessing the prevalence of AO in two Swiss children groups according to five different WC-based definitions, ranging from 1.8% to 55.5% [21]. The high variability in AO prevalence we found according to different charts may depend on several reasons.

Firstly, we used cut-offs that were calculated in children from different areas in the world, but the prevalence of AO in different countries may vary widely and percentile-charts are strongly influenced by the population status in a particular moment. A systematic review analysing AO in adolescents reported a high variability in AO prevalence both in developing and in developed countries, ranging from 3.8% to 51.7% [22]. Therefore, the use of a cut-off calculated in a population with a higher or lower prevalence of AO, may lead to an under- or over-estimate of the real AO prevalence, respectively. All the WC charts included in our paper were calculated analysing both overweight/obese and normal-weight subjects. Very recently, the IDEFICS Consortium published WC percentiles for the paediatric European population aged 2.0–10.9 years that conversely were built by including only normal-weight subjects. They stated that as reference values are expected to reflect the biological variation in a disease free population, it was a logical method to exclude unhealthy subjects (in this case underweight, overweight and obese individuals) from the analysis group for the creation of the charts [23].

Reference percentiles based on nationally representative samples do not already exist for all nations.

And not all the currently used cut-offs are fully representative of a national population. Out of the nine WC charts we used, two are usually referred to as Italian and Turkish charts even if they are calculated from children living in very limited areas [10,16]. When analysing the prevalence of AO separately in our Northern and Southern Italy populations, we found even higher discrepancy according to the nine used WC cut-offs and wide differences between the two areas of the same country, with values ranging from 2.4% to 35.7% in Northern Italy children and from 15.1% to 84.2% in Southern ones. In particular, the lower prevalence of AO found using the Italian reference may be due to the fact that the cited cut-offs by Zannolli et al. [10] were calculated from children living in a limited area around Pescara, a town in Abruzzo region, in Central Italy, where a high prevalence of overweight/obesity was reported [24]. It could lead to an improper underestimation of AO prevalence especially in children from regions with a lower rate of overweight/obesity, such as Piemonte region.

Actually, this discrepancy could not be overcome by regionally assessed cut-offs that would underestimate the real prevalence of AO in children from Southern Italy, where a higher prevalence of overweight/obesity was found [25]. Moreover, there are many people in the world of mixed ethnicity. One step forward might be to identify international WC cut-offs, as Cole et al. have already done with BMI for the diagnosis of overweight and obesity in paediatric age [19,26].

However, the key point is not to implement the role of particular and regional characteristics of different samples, but to develop risk-weighted cut-offs. WC is universally used to define body fatness. The classification of body fatness would ideally be based on the risk of future diseases. Because longitudinal data from pediatric age to adulthood are still lacking, some Authors have based cut-off points for higher body fatness among children on cross-sectional associations with metabolic risk factors by using ROC curves. They used skinfold thickness measurements [27–29] and one of them—on a relatively small prepubertal population—also WC [29]. However, the cut-off points of body fatness drew in these studies did not vary by age, and as a consequence, they could not account for the changes in body fatness occurring during linear growth.

Moreover, ethnicity, instead of nationality, should be considered for fixed WC cut-offs also in children, as pointed by the International Diabetes Federation that differentiates fixed WC cut-offs in adults for Europids, Asians and Japanese, on the basis of data linking WC to several cardiovascular risk factors in different populations [30].

Secondly, an important rate of variability in our AO prevalence may lay on the site of WC measurement used. It has to be pointed out that WC measurement in our populations was performed at just above the uppermost lateral border of the right ilium. And out of the nine selected WC charts, four used the above-mentioned site [9–12], five measured WC at the midpoint between the lowest rib and the iliac crest [13–17]. Having measured WC only in one anatomic point could appear as a limitation. Actually the aim of our study was not to compare AO prevalence according to different WC charts, but to assess how much it could vary using different cut-offs, even more if measured in different anatomic points. If WC had been measured also at the midpoint between the lowest rib and the iliac crest, each of the nine WC cut-offs should have been applied appropriately to the measure taken in the corresponding anatomic site. Therefore, conclusions about AO prevalence variability according to different WC measurement sites could have been drawn. In literature, many studies measured WC in their populations in an anatomic point but calculated AO prevalence using WC cut-offs determined in a different anatomic point [31–34]. Even more, other studies did not report in Methods section the specific site of their WC measurement [35,36]. Such inconsistency could lead to an improper estimate of AO prevalence, since it varies widely according to this methodological issue [37]. In adults, a high variability has been reported in WC measurement sites in the review by Wang et al [38], including 61 studies using different WC measurement methods, categorized into four groups: midway between the bottom of the lower rib and the top of the iliac crest; at the umbilicus; at the narrowest point between the umbilicus and xiphoid process; unspecified or other methods. An even higher heterogeneity has been reported in the systematic review by de Moraes et al, including 29 studies about AO prevalence in adolescents, estimated by the use of fourteen different cut-off anatomic points [22].

International agreement about the measurement site is therefore required in order to facilitate comparisons among studies evaluating AO and metabolic syndrome prevalence. In particular, the standardized anatomic point of WC measurement should be chosen in order to be the best predictor of adverse cardiometabolic outcomes. The sensitivity of WC sites to changes in subcutaneous abdominal and visceral fat distribution, linked to metabolic risk factors, should be established by measuring WC at several sites during longitudinal studies as Johnson et al. propose [39].

Thirdly, a further source of variability may be the statistical method used to calculate WC cut-offs. Only six of the references we considered used the LMS method [12–17], which is the most adequate to develop percentiles of anthropometric measures for children and adolescents. The LMS method enables normalised growth centile standards to be developed, and deals generally with skewness which might be present in the distribution of the WC measurements [40].

The detection of such a high variability in AO prevalence in our population according to different WC cut-offs prompted us to suggest the need for pragmatic fixed and age-sex-stratified WC cut-points above which the occurrence of clustered cardio-metabolic events increases, as already performed in adults [30,41,42]. Up to now, a similar approach has been developed in children for BMI [43]. Therefore, we suggest that risk-weighted cut-offs should be calculated by gender and age, in order to provide clinicians with an easy-to-use tool to identify children at risk for actual and future cardio-metabolic complications to whom specific and prompt health interventions should be addressed. International efforts and consensus are needed to plan future longitudinal studies on metabolic and cardiovascular outcomes. Cross-sectional associations of age and gender WC cut-offs with metabolic risk factors could be a good compromise waiting these longitudinal studies. In this perspective, a single anthropometric measurement may select children deserving of laboratory and instrumental investigations and of dietary and life-style improvements.

## Conclusions

In conclusion, if risk-weighted WC cut-offs measured in a standardized anatomic site and calculated by the appropriate method would be developed, WC measurement could be a simple clinical tool to identify children at high risk of cardio-metabolic complications to whom specific and prompt health interventions should be addressed.
